# Modified Chitosan-Based Hemostatic Dressings Incorporating Heparin-Loaded Nanoparticles for Enhanced Hemostatic Activity

**DOI:** 10.3390/pharmaceutics18030373

**Published:** 2026-03-18

**Authors:** Despoina Meimaroglou, Evi Christodoulou, Rizos Evangelos Bikiaris, Ioanna Koumentakou, Michiel Jan Noordam, Amalia Oikonomou, Ioannis Taitzoglou, Ioannis Tsamesidis, Eleana Kontonasaki, Zoi Terzopoulou, Lysimachos G. Papazoglou, George Z. Kyzas, Dimitrios N. Bikiaris

**Affiliations:** 1 Laboratory of Polymer and Colors Chemistry and Technology, Department of Chemistry, Aristotle University of Thessaloniki, 54124 Thessaloniki, Greece; despinameimar@gmail.com (D.M.); mjnoordam@gmail.com (M.J.N.); 2 Hephaestus Laboratory, School of Chemistry, Faculty of Sciences, Democritus University of Thrace, 65404 Kavala, Greece; rizosbikiaris@gmail.com (R.E.B.); iwanna.koumentakou@gmail.com (I.K.); kyzas@chem.duth.gr (G.Z.K.); 3 Department of Clinical Sciences, School of Veterinary Medicine, Aristotle University of Thessaloniki, 54627 Thessaloniki, Greece; amaliaoikonomou.96@gmail.com (A.O.); makdvm@vet.auth.gr (L.G.P.); 4 Laboratory of Physiology, School of Veterinary Medicine, Aristotle University of Thessaloniki, 54124 Thessaloniki, Greece; jotai@vet.auth.gr; 5 Department of Prosthodontics, Faculty of Dentistry, School of Health Sciences, Aristotle University of Thessaloniki, 54124 Thessaloniki, Greece; johntsame@gmail.com (I.T.); kont@dent.auth.gr (E.K.); 6 Laboratory of Industrial Chemistry, Department of Chemistry, University of Ioannina, 45110 Ioannina, Greece; terzoi@uoi.gr

**Keywords:** hemostatic wound dressings, bleeding control, chitosan, heparin (Hep), polymer nanoparticles

## Abstract

**Background/Objectives**: Achieving effective hemostasis is a vital step in wound healing, particularly in cases of severe bleeding caused by surgical procedures or trauma. This study focuses on the development of chitosan-based dressings enriched with Heparin (hep)-loaded poly(butylene succinate) (PBSu) nanoparticles to combine hemostatic and anticoagulant properties. **Methods**: Chitosan, a biocompatible and biodegradable carbohydrate with inherent antibacterial and hemostatic properties, was chemically modified with 2-(N-morpholino)ethanesulfonic acid (MES) and 2-acrylamido-2-methylpropane sulfonic acid (AMPS) to enhance its swelling ability and hemostatic activity. PBSu nanoparticles were synthesized using an oil-in-water emulsification method and loaded with Hep to achieve controlled anticoagulant release. The dressings of the modified chitosan derivatives with the nanoparticles which were systematically characterized for morphology, chemical structure, swelling ability, loading capacity, and Hep release kinetics. **Results**: This dual-function system is designed to decouple local surface hemostasis from thrombotic processes: the chitosan matrix provides rapid topical hemostasis, while controlled heparin release from the nanoparticles aims to modulate excessive fibrin deposition, support microvascular perfusion, and exploit the pro-healing benefits of low-dose heparin reported in advanced wound dressings, particularly in high-risk or thrombotic-prone patients. In vitro and in vivo studies demonstrated their potential for promoting rapid hemostasis. **Conclusions**: These findings suggest that the integration of modified chitosan and Hep-loaded nanoparticles is a promising strategy for advancing wound care and hemostatic technologies.

## 1. Introduction

Hemostasis is a critical phase of the wound healing process following surgical intervention or acute trauma, during which bleeding is controlled and a provisional matrix forms to protect the injury site [1]. This response proceeds through three major stages: vasoconstriction, formation of a platelet plug, and activation of the coagulation cascade, ultimately resulting in the development of a stable fibrin clot [2,3].

Current research seeks to develop hemostatic materials that are highly biocompatible and capable of rapidly arresting both arterial and venous bleeding. These materials must function without thermal effects, conform to irregular wound geometries, and allow for straightforward application and removal without leaving residues. They should also remain stable under extreme environmental conditions and be cost-effective for widespread medical use [4,5]. While several commercial hemostatic products, such as kaolin-based gauze (Combat Gauze), oxidized regenerated cellulose (Surgicel, Oxycel), fibrin/thrombin patches (TachoSil, Evarrest), and gelatin-based dressings (Spongostan) are available, they may fall short in managing severe hemorrhage and some pose issues related to cost and biocompatibility [6,7,8].

Chitosan (CS), a naturally derived cationic polysaccharide obtained from the deacetylation of chitin, has emerged as a promising material for next-generation hemostatic products. CS is renewable, biodegradable, non-toxic, and exhibits intrinsic bactericidal activity [9]. Its cationic nature promotes hemostasis via electrostatic interactions with negatively charged blood components, including erythrocytes and platelets, thereby accelerating clot formation [10,11,12]. These features have led to the development and commercialization of CS-based wound dressings such as ChitoGauze, Celox Gauze, Mini-Dressing, HemCon, Trauma Gauze, and ChitoFlex [5]. Despite their advantages, certain limitations persist. For example, some dressings can induce hypersensitivity reactions, manifesting as urticaria, angioedema, fever, or lymphadenopathy, while others may be too rigid or brittle for use in complex wound geometries or difficult-to-access bleeding sites [13,14].

Ongoing research aims to develop low-cost, biocompatible, and fast-acting hemostatic materials based on CS [15]. For example, CS has been incorporated with various synthetic polymers, such as poly(vinyl alcohol) (PVA), to create hemostatic compounds with desirable chemical, mechanical, and physical properties, such as fast swelling [5]. Hemostatic products that can form gels upon contact with water and blood, without dissolving, offer several benefits. These gels can create an active coating that protects the injured area from germs, absorbs and retains moisture within its structure, eliminates excess exudates from the wound surface, and supports proper gaseous exchange [16]. CS can form such hydrogels via ionic cross-linking, offering tuneable structural and functional properties.

In a previous study of our group, we incorporated inorganic salts and levofloxacin into CS-based dressings to improve their hemostatic and antibacterial efficacy [2]. Herein, we aimed to fabricate CS-based wound dressings with improved swelling and hemostasis. On one hand, adding sulfonic groups on chitosan can improve the antimicrobial activity due to zwitterionic effects, and on the other hand they can improve hydrophilicity, indirectly enhancing blood absorption and clot formation. To achieve this, and based on previous work by our group where we have already demonstrated the antimicrobial activity of CS-AMPS, CS was modified with 2-acrylamido-2-methylpropane sulfonic acid (AMPS), which is also known for its antibacterial activity against both Gram-positive and Gram-negative bacteria [17,18], and with 2-(N-morpholino)ethanesulfonic acid (MES). The AMPS-modified chitosan, bearing strong anionic sulfonate groups, enhances hydrophilicity and fluid absorption, which leads to rapid plasma uptake and concentration of erythrocytes, platelets, and clotting proteins at the wound interface, thereby accelerating fibrin formation and clot initiation. On the other hand, MES-modified chitosan features a zwitterionic balance of charge in its morpholino ring and sulfonate moiety, which induces a hydrated, non-fouling surface. This zwitterionic hydration layer helps localize clot formation to the wound site and minimizes unintended platelet or protein activation away from the target, improving in vivo hemocompatibility.

Additionally, to regulate clotting and prevent thrombotic complications, Hep, an anticoagulant widely used in medical practice, was encapsulated in poly(butylene succinate) (PBSu) nanoparticles (NPs). PBSu nanoparticles were loaded with Hep to achieve controlled anticoagulant release that does not interfere with the hemostatic efficacy of the dressing. The rationale for this dual functionality lies in the synergetic balance between rapid clot induction by the modified chitosan matrix and the slow release of Hep to prevent excessive clot propagation. Chitosan, a well-established pro-hemostatic biopolymer, facilitates immediate clotting through electrostatic interactions with blood cells and proteins, even under coagulopathic or anticoagulated conditions. Importantly, our in vivo results confirm that clot formation is not compromised, as we observed rapid hemorrhage cessation alongside well-contained clot architecture. Upon being modified with AMPS and MES, chitosan increases hydrophilicity and water uptake and preserves the cationic character of the chitosan backbone, thereby improving blood absorption and contact while maintaining strong electrostatic interactions with blood cells; together these effects enhance platelet capture, erythrocyte aggregation, and local fibrin network formation compared with neat chitosan [19,20,21,22]. PBSu, an aliphatic polyester, was selected for its biodegradability, renewability, and favourable physicochemical properties, including tuneable degradation rate. Unlike poly(lactic-co-glycolic acid) (PLGA), which may generate acidic degradation products affecting local pH and tissue compatibility, or polycaprolactone (PCL), which degrades too slowly, PBSu provides neutral, gradual degradation, ideal for sustained Hep release. Multiple in vitro cytotoxicity studies show no toxicity and favourable tissue response when implanted in rats for up to nine months. Further in vitro cytocompatibility assays confirm minimal adverse effects on cell morphology, proliferation, or function—supporting its suitability in biomedical applications. Collectively, these properties justify the selection of PBSu over PLGA or PCL for a hemostatic sponge system requiring controlled, biocompatible delivery of Hep [23,24,25].

This study aims to prepare and characterize multifunctional CS-based dressings with enhanced hemostatic activity, containing components that have inherent antibacterial and anticoagulant properties. We performed a comprehensive evaluation of the materials’ morphology, chemical composition, swelling behaviour, Hep loading capacity, and release kinetics. Moreover, the hemostatic potential of these materials was assessed in vitro and in vivo. Importantly, the novelty of this work lies in the development of a unique multifunctional system that integrates sulfonic acid-functionalized chitosan derivatives with Hep-loaded polyester nanoparticles, offering a combined approach to hemostasis, antibacterial protection, and antithrombotic activity. Collectively, these investigations aim to advance the design of effective wound dressings that promote safe and effective hemostasis while addressing infection risk and thrombotic complications.

## 2. Materials and Methods

### 2.1. Materials

Medium molecular weight chitosan (CS) with a molecular weight of 20,000 g/mol and degree of deacetylation of 80% was purchased from Kraeber & Co GmbH (Ellerbek, Germany). 2-(N-morpholino)ethanesulfonic acid (MES) (low moisture content, ≥99%), 2-acrylamido-2-methylpropane sulfonic acid (AMPS) (99%), N-(3-Dimethylaminopropyl)-N′-ethylcarbodiimide (EDC, ≥97.0%), polyvinyl alcohol (PVA, Mw 89,000–98,000, 99+% hydrolyzed), and potassium persulphate (99.99% trace metals basis) were purchased from Sigma-Aldrich (Darmstadt, Germany). Potassium persulfate (KPS, K_2_S_2_O_8_, purum ≥ 99.0%) was purchased from Merck (Darmstadt, Germany). Hep sodium 1% in Fitalite was supplied by Fagron Hellas (Trikala, Greece). All other reagents used were of analytical grade.

### 2.2. Methods

#### 2.2.1. Synthesis of Modified CS Derivatives

To prepare MES-modified chitosan (CS-MES), 1 g of CS was dissolved in 100 mL of 1% (*v*/*v*) aqueous acetic acid with stirring at room temperature (RT) for 2–3 h. Separately, 0.5 g of MES was dissolved in 20 mL deionized water, and the pH adjusted to 5.0 with 0.1 M NaOH. Then, 0.5 g of EDC was added to the MES solution and stirred for 30 min at RT to activate the carboxyl groups. The activated MES-EDC solution was slowly added to the CS solution, followed by stirring at 50 °C for 6 h. The reaction mixture was purified by dialysis against deionized water using a 10 kDa MWCO membrane for 48 h to remove unreacted reagents and byproducts. The purified solution was freeze-dried to yield CS-MES as a porous dressing.

**Figure 1:**
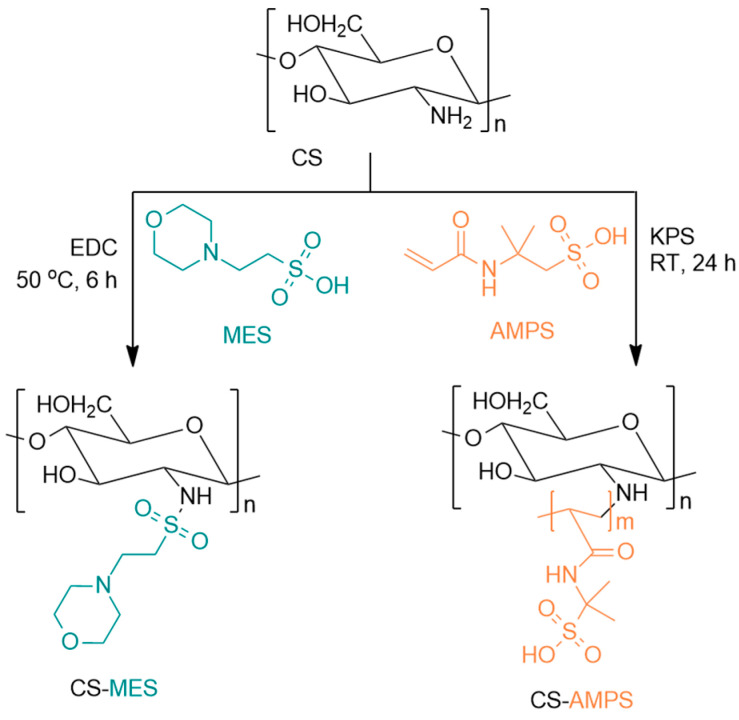
Modifications of CS using MES and AMPS.

AMPS-modified CS (CS-AMPS) was synthesized by dissolving 10 g (2.5% *w*/*v*) of CS in 400 mL of 2% *v*/*v* acetic acid aqueous solution under stirring at RT for 24 h. After complete dissolution, 1.48 g of AMPS and 0.03748 g KPS were added to the mixture. The grafting reaction was carried out at 60 °C for 6 h under a nitrogen atmosphere with continuous magnetic stirring, leading to the formation of a viscous liquid. The product was then frozen and freeze-dried at −56 °C. The final product was further purified by Soxhlet extraction using acetone as an eluent to remove any unreacted monomer. An overview of the modification reactions is presented in Figure 1.

#### 2.2.2. Preparation of PBSu and Hep-Loaded PBSu NPs

PBSu NPs were prepared using the oil-in-water (O/W) emulsification/solvent evaporation method, with poly(vinyl alcohol) (PVA) as a nonionic polymeric emulsifier. The PBSu with Mn = 40,000 g/mol and Mw = 65,000 g/mol was previously synthesized by our group, according to well-established methods [22,23]. Briefly, 333.33 mg PBSu were dissolved in 9 mL of dichloromethane. 0.02 g of Hep was introduced and stirred gently for 1 h. Subsequently, 20 mL of an aqueous PVA solution (1% *w*/*v*) was added dropwise in the organic solution. The mixtures were stirred for 2 h to ensure complete solvent evaporation. Probe sonication at X rpm was performed twice for 1 min, leading to the formation of an oil-in-water emulsion (o/w). NPs were collected by centrifugation at 11,000× *g* rcf for 10 min. The obtained NPs were washed once with water, and the resulting aqueous nano-suspension was lyophilized using a Scavnac freeze-drier system (Coolsafe 110-4 Pro, Labogene, Allerød, Denmark) at −56 °C for 24 h, yielding the final dried NPs. The resulting PBSu NPs exhibited a polydispersity index (PDI) of 0.4, as determined by dynamic light scattering, indicating a narrow particle size distribution.

#### 2.2.3. Preparation of NPs-Enriched CS Dressings

To prepare the NPs-enriched CS dressings, the Hep-loaded PBSu NPs were dispersed in a 1% (*v*/*v*) aqueous acetic acid solution of each CS derivative, with an NPs concentration of 2% (*w*/*v*) in the CS solutions. To ensure a uniform distribution of the NPs within the CS matrix, the dispersion process was enhanced via probe sonication, conducted twice for 1 min at an amplitude of 30%.

Next, the mixtures underwent a controlled freeze-drying process. Initially, the samples were frozen at −80 °C for 24 h to solidify the matrix. Afterwards, the frozen samples were placed in the freeze-dryer system under vacuum conditions for 48 h to remove the aqueous solvent, resulting in the samples obtaining a porous powder form. The obtained powders were gently milled and sieved to achieve a consistent particle size. Finally, the CS dressings were stored in airtight containers under desiccated conditions to prevent moisture absorption until further characterization and use.

### 2.3. Characterization of the Prepared Materials

#### 2.3.1. Molecular Weight Determination

The chitosans’ molecular weight measurements were determined through the intrinsic viscosity according to the bibliography [26,27]. The intrinsic viscosity [*η*] was determined at 25 °C using a Type 2C Ubbelohde viscometer (Witeg Labortechnik GmbH, Wertheim am Main, Germany). Each chitosan sample was dissolved in an aqueous solution containing 0.1 M acetic acid and 0.02 M NaCl to a final concentration of 1% *w*/*v*. The intrinsic viscosity of the resulting solutions was calculated using the following equations:

(1) $$\eta_{r}=\frac{t}{t^{0}}$$

(2) $$\eta_{sp}=\eta_{r}-1$$

(3) $$\eta_{red}=\frac{\eta_{sp}}{c}$$

(4) $$\left[\eta \right]=\underset{c\to 0}{\lim}\left(\eta_{red}\right),$$

where *c* is the concentration of the solution, *t* the flow time of solution, and *t*_0_ the flow time of the solvent.

The molecular weight was measured according to the equation:

(5) $$\left[\eta \right]=3.04\times 10^{-5}M_{v}^{1.26},$$

#### 2.3.2. Fourier-Transformed Infrared Spectroscopy (FT-IR)

The chemical structure of the samples was determined by FT-IR spectroscopy. FT-IR spectra were obtained using an FTIR-2000 (Perkin Elmer, Dresden, Germany) with KBr discs. The spectra were collected in the range from 4000 to 400 cm^−1^ at a resolution of 4 cm^−1^ (32 co-added scans).

#### 2.3.3. X-Ray Diffractometry (XRD)

X-ray powder diffraction (XRD) analysis was performed using an X-ray diffractometer (Rigaku-Miniflex II, Tokyo, Japan) equipped with a CuKα radiation source (wavelength: 0.15405 nm). The measurements were conducted under operated conditions of 30 kV and 15 mA to obtain well resolved diffraction peaks with sufficient intensity. Diffraction patterns were collected within a 2θ range of 5–40°, using a scanning speed of 1° min^−1^ and a step interval of 0.05°.

#### 2.3.4. Particle Size and ζ-Potential Estimation (DLS)

Particle size and ζ-potential of the nanoparticles (NPs) were measured via dynamic light scattering (DLS) on a Zetasizer Nano ZS (ZEN3600, Malvern Instruments, Malvern, Worcestershire, UK) instrument equipped with a 532 nm laser. Measurements were conducted at 90° scattering angle and 25 °C. Suspensions were prepared in 10^−4^ M NaCl aqueous solution after sonication at 25 °C, with analyses performed in triplicate.

#### 2.3.5. Scanning Electron Microscopy (SEM)

Nanoparticle morphology and size were examined by scanning electron microscopy (SEM). Samples were coated with a thin carbon layer to enhance electron beam conductivity prior to imaging on a JEOL JMS-840A microscope (Jeol Ltd., Akishima, Japan). Imaging used an accelerating voltage of 10 kV, probe current of 45 nA, and counting time of 60 s.

#### 2.3.6. Swelling Capacity and Stability

The stability of the CS dressings was examined in an acid solution (pH = 5.5) and simulated body fluid (SBF, pH = 7.4). The acid solution was prepared using distilled water and ortho-phosphoric acid, while the SBF solution was prepared based on a previously described method [28]. A total of 0.5 g of each CS dressing was immersed in 10 mL of the respective solutions. The physical stabilities of the dressings were observed after 10, 20, 30, 60 and 120 min under ambient conditions.

Swelling ratio and water content were assessed using the same acid and SBF solutions. Each sample was initially weighed (W_1_) and then immersed in the respective solution. At each predetermined time point (10, 20, 30, 60 and 120 min), the dressings were taken out of the solutions, excess surface liquid was wiped off via filter paper and, reweighted (W_2_). The swelling ratio and water content were calculated at each time interval according to the following equations [29]:

(6) $$Swelling\;ratio\;(\%)=\frac{\left(W_{2}-W_{1}\right)}{W_{1}}\times 100$$

(7) $$Water\;content\;(\%)=\frac{\left(W_{2}-W_{1}\right)}{W_{2}}\times 100$$

The kinetics of water loss between the maximum water content and the equilibrium was examined via a first-order kinetic process according to the following equation [30]:

(8) $$M\left(t\right)=M_{eq}+\left(M_{max}-M_{eq}\right)\times e^{-kt}$$

where *M*(*t*) and *M_eq_* are the masses of water adsorbed at time t and equilibrium, respectively; *M_max_* is the maximum mass of water adsorbed and k is the water adsorption rate constant. The procedure was repeated twice.

#### 2.3.7. In Vitro Release of Hep

The total amount of encapsulated Hep was quantified using a modified toluidine blue (TB) colorimetric assay, based on previous methods [31,32]. A 0.005% (*w*/*v*) TB solution was prepared in 0.01 N HCl containing 0.2% (*w*/*v*) NaCl. A standard Hep solution was prepared by dissolving 20 mg of Hep in 20 mL of deionized water and subsequently diluted to obtain a series of standard solutions of known concentrations. For the loading assay, a known weight of lyophilized nanoparticles was redispersed in simulated body fluid (SBF), under gentle stirring, to release the encapsulated Hep into the aqueous phase. A total of 1.5 mL of the TB solution was mixed with 1.5 mL of either the standard Hep solution or the NP dispersion in SBF. The mixtures were vortexed for 30 s, followed by the addition of 3 mL n-hexane and the mixtures were vortexed again for 30s to extract excess dye. The aqueous phase, containing the TB-Hep complex, was collected and measured spectrophotometrically at 630 nm using a Shimadzu UV-Vis1700 (Shimadzu, Kyoto, Japan) within 30 min. Samples were diluted with SBF, if needed, to ensure readings fell within the linear calibration range.

The loading of Hep was calculated using the following equation:

(9) $$Loading\;(\%)=\frac{amount\;of\;total\;entrapped\;drug}{total\;nanoparticle\;weight}\times 100$$

The release profile of Hep from the PBSu nanoparticles and the CS-based matrices was evaluated using a DISTEK Dissolution Apparatus Evolution 4300 (2100C) (North Brunswick, NJ, USA), equipped with USP Apparatus II (paddle method). Approximately 10 mg of each sample (equivalent to a known Hep amount based on loading data) was placed inside dialysis bags (MWCO: 10 kDa), which were then suspended in 300 mL of SBF (pH 7.4) maintained at 37 ± 0.5 °C. The paddle rotation speed was set to 50 rpm. At predetermined intervals (e.g., 0.5, 1, 2, 4, 8, 12, and 24 h), 2 mL aliquots were withdrawn and replaced with an equal volume of fresh pre-warmed SBF to maintain sink conditions. Samples were analyzed using the TB assay described above.

The raw (non-normalized) Hep release data were fitted using the following modified Weibull function, which accounts for a delayed onset, a finite asymptotic release value and a non-exponential release profile:

(10) $$Q\left(x\right)=Q_{\infty }[1-\exp\left(-{\left(\frac{x-T}{\alpha }\right)}^{\beta }\right)]$$

In this model *Q*(*x*) is the cumulative amount of Hep released at time *x*, *Q*∞ is the maximum (asymptotic) release amount, *T* is the time delay before release begins, *α* is the scale parameter, and *β* is the shape parameter.

To compare the Hep release profiles across different samples, the data were normalized and modelled using the standard Weibull cumulative distribution function (CDF):

(11) $$F\left(x\right)=1-exp(-({\frac{x}{\alpha })}^{\beta })$$

In this model, *F*(*x*) represents the normalized fraction of Hep released at time *x*, with *α* and *β* defined as above.

#### 2.3.8. Biocompatibility Evaluation by MTT Assay

The biocompatibility was assessed using the MTT assay (3-(4,5-dimethylthiazol-2-yl)-2,5-diphenyltetrazolium bromide), which evaluates cell metabolic activity as an indirect measure of cell viability and proliferation. Equal amounts of each sample were disinfected in 70% ethanol overnight, rinsed with sterile PBS, and equilibrated for 24 h in Dulbecco’s Modified Eagle Medium (DMEM, #L0104-500, BIOWEST, Nuaillé, France) supplemented with 10% fetal bovine serum (FBS) and penicillin–streptomycin (BIOWEST, #L0018-100). Normal human dermal fibroblasts (HDFs) were seeded in 96-well culture plates (CELLSTAR^®^, #655180, Greiner Bio-One, Kremsmünster, Austria) and cultured until ~80% confluence, which was considered as time zero. Equal-sized scaffold/nanoparticle samples were then placed directly onto the cells, and after 24 h exposure, the MTT assay was performed by incubating the cells with 0.1 mg/mL MTT solution in complete DMEM for 4 h at 37 °C under 5% CO_2_, allowing the formation of intracellular purple formazan crystals. The MTT solution was then removed, dimethyl sulfoxide (DMSO) was added for 30 min to dissolve the crystals, and absorbance was measured. Human dermal fibroblasts cultured on plastic served as positive controls. The experiments were conducted in triplicate, and results were expressed as mean ± SD; statistical significance between groups was determined using Student’s *t*-test with *p* < 0.05 considered significant.

#### 2.3.9. Evaluation of Antibacterial Activity

The in vitro antibacterial activity was evaluated using a standard agar diffusion assay. Briefly, sterile Petri dishes containing nutrient agar were inoculated with bacterial suspensions of *Escherichia coli* (Gram-negative) and *Staphylococcus aureus* (Gram-positive) to achieve uniform lawn growth. Sterilized discs impregnated with the tested materials (CS and CS-AMPS) were carefully placed on the agar surface, and the plates were incubated at 37 °C for 24 h. Following incubation, the antibacterial activity was assessed by measuring the diameter of the inhibition zones formed around each disc, which reflects the ability of the material to diffuse into the agar and suppress bacterial growth.

#### 2.3.10. Assessment of Blood Clotting Time

Clotting time (CT) was estimated based on a previously described method [2]. Blood was collected from healthy volunteers under informed consent following protocol approved as per Helsinki guidelines. Briefly, 10 mg of each CS-derivative was placed into individual Eppendorf tubes and incubated in a water bath at 37 °C for 10 min. Next, 340 μL of whole blood containing 3.8% sodium citrate, an anticoagulant, and 20 μL of 0.2 M CaCl_2_ were added to each tube, followed by incubation in a water bath at 37 °C. The tubes were inverted every 10 s held in position for 1 s before reverting. CT was recorded as time in seconds when no blood flow was observed after inversion. The experiment performed in triplicate, and a sample without any CS-derivative served as a control.

#### 2.3.11. Evaluation of Hemostatic Ability

**Figure 2:**
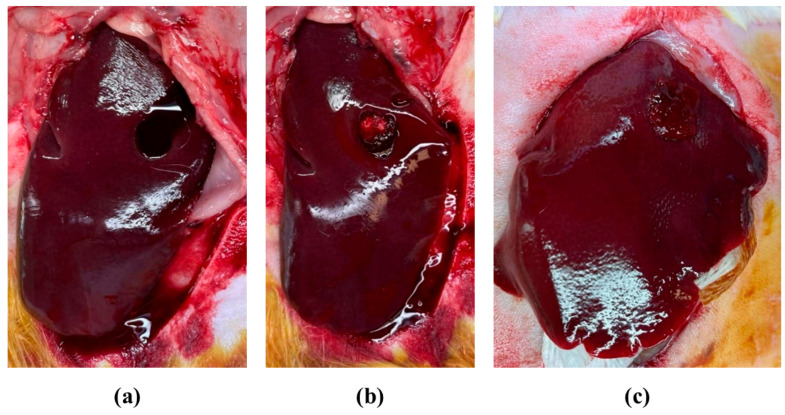
Images of (**a**) untreated bleeding wound, (**b**) wound treated with CS-MES + NPs dressings before saturation, and (**c**) wound treated with CS-MES + NPs dressing after saturation. Photograph courtesy of Amalia Oikonomou. Copyright 2025.

The experimental protocol complied with all local legislation regarding laboratory animal safety and welfare and was approved by the State Veterinary Services. The research protocol received the required permission from the Veterinary Directorate of the Region of Central Macedonia with protocol number 465097(2106). The procedure was based on a previously described method [33]. Twenty-two male New Zealand White rabbits (3.3 ± 0.5 kg) were purchased from Giannarelis Farm (Gazoro, Serres, Greece; registration number EL-62-RAB) and allocated in two equal groups (*n* = 11), designated as Group A and Group B. All animals were sedated via intramuscular injection of medetomidine (0.5 mg/kg; Dorbene Vet 1 mg/mL, Zoetis Hellas SA, Athens, Greece), and general anesthesia was induced via intramuscular administration of ketamine (35 mg/kg; Ketamidor 100 mg/mL, Richter Pharma AG, Wels, Austria) [34,35]. Anesthetic depth and vital signs, namely reflexes, respiratory rate and cardiac function, were continuously monitored throughout the procedure. Following surgical preparation of the ventral abdomen, a midline laparotomy was performed using a No. 10 scalpel and the liver was isolated from the abdominal cavity using laparotomy dressings. Two cylindrical wounds (8 mm in diameter) were created on the visceral surface of each lateral live lobe using a dermal biopsy punch, with approximately 2 cm spacing between both wounds to avoid interaction (Figure 2a). The excised tissue was completely resected.

In Group A, a CS-MES + NPs dressing was randomly applied to one wound of each lobe, and a cotton dressing (Cotton Rolls, Ø 0.8 cm/Euronda Monoart, Vicenza, Italy) to the other (Figure 2b). In Group B CS-AMPS + NPs and CS + NPs dressings were applied in a similar manner. Dressing placement was randomized using a random number generator (Random.org, Randomness and Integrity Services Ltd., Dublin, Ireland). All dressings were cylindrical, measuring 8 mm in diameter and 0.5 cm length, and no additional pressure was applied during placement. Non-woven gauges (Non-woven swabs, 5 × 5 cm, ABENA, Aabenraa, Denmark) were used to absorb blood and plasma from the bleeding wounds.

At the onset of bleeding, hemorrhage was classified as mild, moderate, or severe. Timing began immediately after dressing placement, with bleeding observations recorded every 15 s. After 10 min, timing ceased, and the materials were removed and weighed (Figure 2c). The difference in weight of the dressings and gauze pads before and after the procedure was recorded as total blood loss (TBL). Successful hemostasis was defined as the cessation of bleeding (blood or plasma flow) within 3 min. Bleeding persisting beyond 10 min was determined a hemostasis failure.

#### 2.3.12. Statistical Analysis

CT data were compared using one-way ANOVA, followed by Tukey’s Honest Significant Difference (HSD) test for post hoc multiple-comparison testing. Hemostasis times and TBL data were analyzed using the Scheirer–Ray–Hare test, as these data exhibited a nonparametric distribution according to the Kolmogorov–Smirnov test. Mann–Whitney U tests were used for post hoc analyses, with Bonferroni correction applied for multiple comparisons. CT is expressed as mean ± SD, whereas hemostasis times and TBL data are expressed as median ± 95% CI. A *p*-value of <0.05 was considered statistically significant. Scheirer–Ray–Hare tests were performed using the “Real Statistics Resource Pack” add-on for Excel, while all other analyses were conducted using IBM SPSS Statistics v28.0.

## 3. Results and Discussion

### 3.1. Structural Properties

#### 3.1.1. Molecular Weight Determination

The viscosity-average molecular weights (Mν) of pure and chemically modified chitosans were determined by intrinsic viscosity measurements, as described in Section 2.3.1. All measurements were performed in triplicate, and the corresponding molecular weight was calculated using Equations (1)–(5). Pure chitosan exhibited a Mν value of approximately 19,000 g/mol. A moderate decrease in molecular weight was observed after functionalization, with CS-MES and CS-AMPS showing Mν values of 16,800 and 17,300 g/mol, respectively, suggesting that the modification reactions did not induce significant polymer chain degradation.

#### 3.1.2. FT-IR Spectroscopy

**Figure 3:**
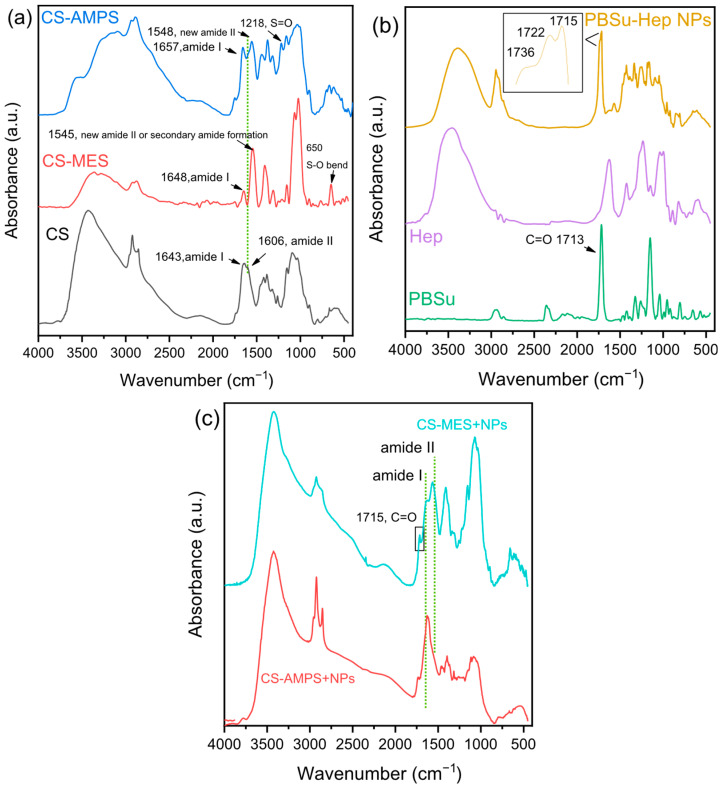
FT-IR spectra of (**a**) CS, CS-MES and CS-AMPS, (**b**) PBSu, Hep and Hep-loaded PBSu NPs, (**c**) Final materials CS-MES-NPs and CS-AMPS-NPs.

The chemical structure of CS and its derivatives was studied with FT-IR, presented in Figure 3a. In the neat CS spectrum, the broad band at 3200–3500 cm^−1^ is attributed to the overlapping hydroxyl O-H and primary amine N-H stretch vibrations, whereas other characteristic bands of amide I are located at 1643 cm^−1^, and amide II at 1606 cm^−1^ [36]. Primary amine vibrations tend to overlap with the amide I bands. The bands at 1155 cm^−1^ (anti-symmetric stretching of the C-O-C bridge), 1090 and 1032 cm^−1^ (skeletal vibrations involving the C-O stretching) are characteristic for any polysaccharide structure [2]. In the spectrum of CS-MES there are notable changes. The amide I band shifted slightly to 1648 cm^−1^ and decreased in intensity, while the amide II band at 1606 cm^−1^ disappeared, suggesting that the primary amino groups of CS were involved in the reaction. A new band emerged at 1545 cm^−1^, which may be attributed to the formation of new amide or sulfonamide linkages. Additionally, a much stronger peak appeared at 1023 cm^−1^ in the CS-MES spectrum, likely arising from C–O or C–N stretching, and possibly influenced by the sulfonic acid and morpholine groups [37,38,39]. A distinct new peak at 650 cm^−1^, characteristic of S–O bending vibrations, further supports the presence of sulfonic acid groups from MES. The broad band at 3200–3500 cm^−1^ significantly decreased in both area and intensity, indicating a reduction in free hydroxyl and amino groups, and a likely decrease in hydrogen bonding due to the introduction of the MES moieties. These spectral changes collectively suggest the successful incorporation of MES into the chitosan backbone.

Notable shifts in the FT-IR spectrum of CS-AMPS were also observed, indicating interactions between the CS and the AMPS molecules. Specifically, the broad band corresponding to the O-H and N-H stretching vibrations split and shifted towards lower wavenumbers, indicating reduction in intra/inter molecular hydrogen bonding caused by the bulky AMPS chains. The amide I band shifted to 1657 cm^−1^ and the amide II band was again affected, since a band at 1556 cm^−1^ emerged related to a new amide band. These changes suggest the formation of new C-N bonds between the amine groups of CS with the aliphatic chain of AMPS [17,40]. Finally, the presence of a sharp peak at 1218 cm^−1^ strongly suggests the presence of S=O symmetric stretching of the sulfonic acid group of AMPS.

The spectra of PBSu, Hep and Hep-loaded PBSu NPs are presented in Figure 3b. PBSu typically exhibits a strong absorption band at around 1713 cm^−1^, which corresponds to the C=O stretching vibration of the ester carbonyl groups present in the polymer backbone. A prominent peak near 2945 cm^−1^ is attributed to the C–H stretching vibrations of the methylene (–CH_2_–) groups found in both the butylene and succinate segments. Additionally, a characteristic band at approximately 1150 cm^−1^ arises from the C–O–C stretching vibrations of the ester linkages within the polymer structure. Hep on the other hand displays characteristic FTIR absorption bands, including a broad peak around 3465 cm^−1^ attributed to O–H stretching vibrations from hydroxyl groups and bound water. The region between 2940 and 2840 cm^−1^ corresponds to C–H stretching vibrations of CH, CH_2_, and CH_3_ groups. A peak near 1626 cm^−1^ is assigned to hydroxyl bending vibrations or amide I (C=O stretching). The band at 1414 cm^−1^ is related to N–H bending vibrations (amide II region). Additionally, the sulfonate groups (–SO_3_^−^) exhibit asymmetric stretching around 1240 cm^−1^ and symmetric stretching near 1032 cm^−1^. After the incorporation of Hep into PBSu NPs, a broad band appeared in the 3200–3500 cm^−1^ region, attributed to the O–H and N–H stretching vibrations from Hep’s hydroxyl and amine groups, suggesting hydrogen-bonding interactions with PBSu. The carbonyl stretching band of PBSu exhibited broadening and splitting into multiple components with shoulders emerging at 1736 and 1722 cm^−1^, and a dominant peak slightly shifted to 1715 cm^−1^ shown in the inset of Figure 3b. This splitting implies possible interactions between Hep’s functional groups and the ester carbonyls of PBSu, potentially due to hydrogen bonding or localized microenvironment changes. Additionally, the appearance of a new band at 1570 cm^−1^ may be attributed to N–H bending or to asymmetric stretching of carboxylate or sulfonate groups from Hep, further supporting its successful incorporation into the nanoparticulate system [41].

Upon addition of Hep-loaded PBSu NPs to CS-MES, small spectral changes confirmed their successful incorporation and interaction. As seen in Figure 3c, the broad O–H/N–H band intensified and shifted from 3360 to 3400 cm^−1^, indicating enhanced hydrogen bonding. A new peak at 1715 cm^−1^ confirmed the presence of PBSu carbonyls. The amide I band shifted from 1648 to 1627 cm^−1^, and the band at 1545 cm^−1^ shifted to 1564 cm^−1^, suggesting further interactions involving N–H or sulfonate groups. Additionally, the C–O/C–N band at 1023 cm^−1^ shifted to 1035 cm^−1^, reflecting changes in the local chemical environment and molecular interactions of the chitosan backbone upon incorporation of NPs.

#### 3.1.3. Crystalline Structure

**Figure 4:**
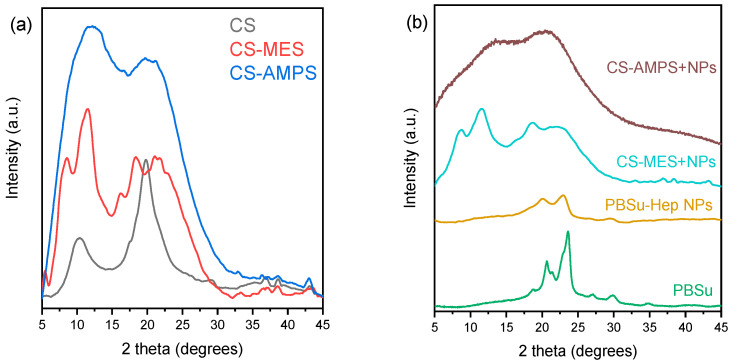
XRD patterns of (**a**) CS, CS-MES and CS-AMPS, (**b**) PBSu, Hep, Hep-loaded PBSu NPs, CS-MES + NPs and CS-AMPS + NPs.

The diffraction patterns of all samples are presented in Figure 4. The two broad diffraction peaks of CS are observed at 2*θ* values of 11° and 19.8°, which are consistent with the values reported in the literature and are related to the extensive inter- and intra-molecular hydrogen bonding of chitosan [42,43,44]. As seen in Figure 4a**,** upon modification, CS-AMPS and CS-MES show significant peak broadening, as well as a shift in the peak at 19.8° towards slightly higher angles, 21.2° for CS-AMPS and 21.5° for CS-MES indicating a decrease In d-spacing and crystallinity. Additionally, new peaks appeared with the incorporation of MES at 2*θ* values of 11.6°, 16.1 and 18.6° that suggest partial crystalline organization of the grafted moieties. Overall, the peak broadening and shifting reflect reduced crystallinity and disrupted hydrogen bonding due to successful functionalization.

PBSu is a polyester that crystallizes easily, as witnessed by its XRD pattern in Figure 4b, showing various sharp diffraction peaks at 19.7°, 21.9°, 22.6° and 28.7° [45,46]. After the encapsulation of Hep, the pattern of PBSu-Hep-NPs showed decreased crystallinity, since the peaks reduced in number and intensity. This amorphization is related to both the disruption of the chain folding of PBSu due to the presence of Hep, and the NP preparation procedure that involved introduction of PVA as well as rapid solvent evaporation that could lead to maintaining the polymer chains in a disordered state. The addition of the NPs in either CS derivatives did not infer significant changes to their crystallinity due to their small amount (2 wt%).

#### 3.1.4. Swelling Ratio and Stability

**Figure 5:**
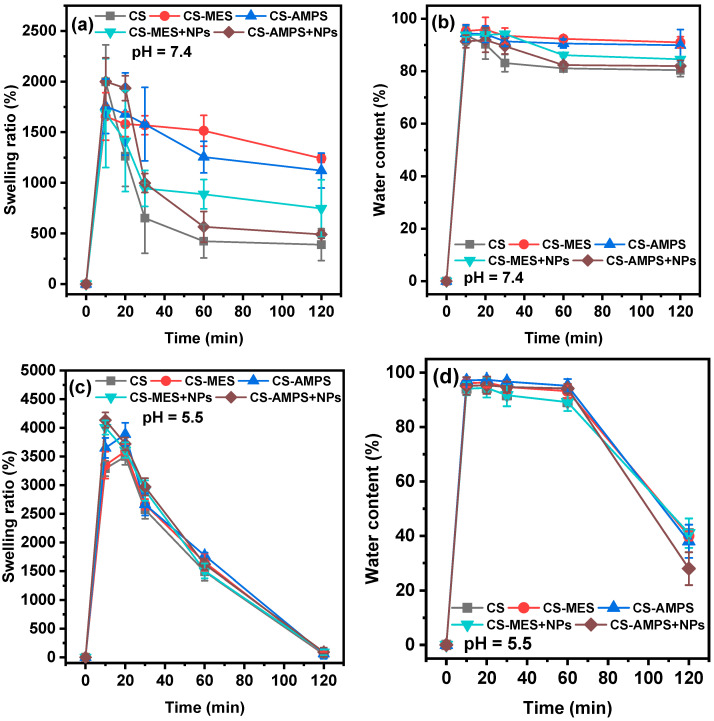
Swelling ratio and water content at (**a**,**b**) pH = 7.4 and (**c**,**d**) pH = 5.5.

Stability, gel formation, and a high water absorption capacity are the desired attributes for hemostatic hydrogels [47]. The effect of the modification and addition of NPs on the swelling behaviour and physical stability of the materials were assessed. The swelling ratio and water content of the dressings at pH = 7.4 and 5.5 are shown in Figure 5.

At pH = 7.4, all wound dressings exhibited their highest swelling ratios and water content at 10 min, with values ranging from 1655% to 2000% and water contents from ~92–98% (Figure 5a,b). After this initial peak, both parameters declined steadily, reaching equilibrium at 120 min with swelling ratios between 392% and 1241% and water contents ~80.5–92.5% (Figure 5a,b). The time-dependent changes in water content were well described by a first-order kinetics model (Equation (8)), with all R^2^ values exceeding 0.90, indicating good model fits (Table 1). A closer examination revealed notable differences between the various wound dressings. All chemically modified samples showed higher swelling ratios and water contents than the neat CS dressing. In particular, CS-MES and CS-AMPS achieved the highest equilibrium swelling ratios (1241% and 1121%, respectively), and correspondingly high water contents of 91.0% and 92.5%. This enhancement was attributed to the introduction of functional groups that increase the hydrophilicity of the polymer network, consequently leading to greater water uptake. While native CS is largely insoluble in neutral pH due to the deprotonated state of its amine groups and extensive inter/intramolecular hydrogen bonding, its functionalization with either MES or AMPS led to improved water uptake, facilitated by both the participation of primary amines in new covalent bonds as well as the presence of sulfonyl or sulfonic groups which are highly hydrophilic in physiological pH [48]. The addition of PBSu-Hep NPs to the modified CS matrices resulted in slightly reduced equilibrium swelling ratios and water contents compared to their unloaded counterparts, though both remained superior to neat CS. This decrease is likely due to hydrophobic nature of PBSu as well as the denser polymer network formed by the interactions of the CS matrix with the NPs, reducing free volume and restricting water penetration.

Quantitatively, the reduction in water content from 10 to 120 min (Water Content Loss) varied among the dressings (Table 1). Neat CS showed the greatest water loss at 13.27%, while CS-MES and CS-AMPS exhibited significantly lower losses at 4.37% and 5.23%, respectively. CS-MES/Hep and CS-AMPS/Hep, had intermediate losses of 9.24% and 9.37%. Corresponding rate constants (k) derived from first-order kinetic modelling also reflected these trends: CS-AMPS exhibited the slowest rate of water loss (k = 0.83 × 10^−3^), followed by CS-MES (k = 1.4 × 10^−3^), indicating their superior water retention capacities while neat CS exhibited the fastest rate (k = 4.6 × 10^−3^) and the NP-loaded variants showed intermediate rates (CS-MES/Hep: 2.7 × 10^−3^; CS-AMPS/Hep: 3.3 × 10^−3^), reflecting the balancing effect of network densification and hydrophilic functionalization.

In a slightly acidic environment (pH = 5.5, close to healthy skin), all materials initially exhibited high swelling ratios after 10 min, ranging between 3290% and 4136% (Figure 5c), which was nearly twice as high as those observed at pH = 7.4 (Figure 5a). Correspondingly, water content ranged between 93.8% and 98.1% (Figure 5d). These swelling ratios and water contents remained relatively stable up to 20 min. In pH = 5.5, both the amine groups of CS and the sulfonate/sulfonyl groups of CS-AMPS and CS-MES are mostly protonated, with also potential zwitterionic behaviour and ionic cross-linking, which results in initially increased water absorption, as counterions from the medium are drawn into the polymeric network. However, in contrast to the water content kinetics at pH = 7.4 where the equilibrium was maintained, at pH = 5.5 all samples deswelled after ~30 min and effectively lost most of their water content after 2 h, suggesting the collapse of the hydrogel network under slightly acidic conditions. This deswelling behaviour and collapse of the hydrogel network is consistent with known limitations of chitosan-based hydrogels, where counterion redistribution and new hydrogen bonding gradually reduce electrostatic repulsion and cause structural compaction; thus, the observed instability at pH 5.5 is an expected phenomenon.

### 3.2. Morphological Examination

DLS analysis revealed differences in particle size between neat PBSu NPs and Hep-loaded PBSu NPs (Table 2). The PBSu NPs had a Z-average of 506 nm with PDI 0.39, indicating a broad and polydisperse particle size distribution. The increase in average particle to ~800 nm size upon Hep encapsulation can be attributed to the successful incorporation of Hep into the NP matrix. Additionally, the broader size distribution of the Hep-loaded PBSu (PDI = 0.58), suggests increased heterogeneity in the particle formation process. Nevertheless, the overall increase in particle size for Hep-loaded NPs aligns with expectations for the encapsulation processes, maintaining their submicron average size.

The micrographs of neat PBSu NPs confirm the successful formation of spherical NPs with well-defined shapes and varying sizes, in agreement with the DLS data. After the encapsulation of Hep, the NPs maintained their spherical shape but had a larger overall particle diameter, which can be attributed to the successful encapsulation of Hep molecules within the NP matrix. The observed increase in NP size can be attributed to molecular interactions that were also evidenced by FTIR. Hydrogen bonding between Hep’s hydroxyl and amine groups and PBSu’s ester carbonyls, as shown by broad O–H/N–H stretching and carbonyl band splitting, suggests strong interfacial binding. The presence of carboxylate or sulfonate groups further supports Hep’s localization at the particle surface. These interactions likely disrupted PVA stabilization during emulsification, reducing interfacial stability and leading to emulsion droplet coalescence and ultimately, larger nanoparticles. Additionally, the particle’s surface was rough, likely due to the hydrophobicity and crystallizability of PBSu which could lead to less stable emulsions.

**Figure 6:**
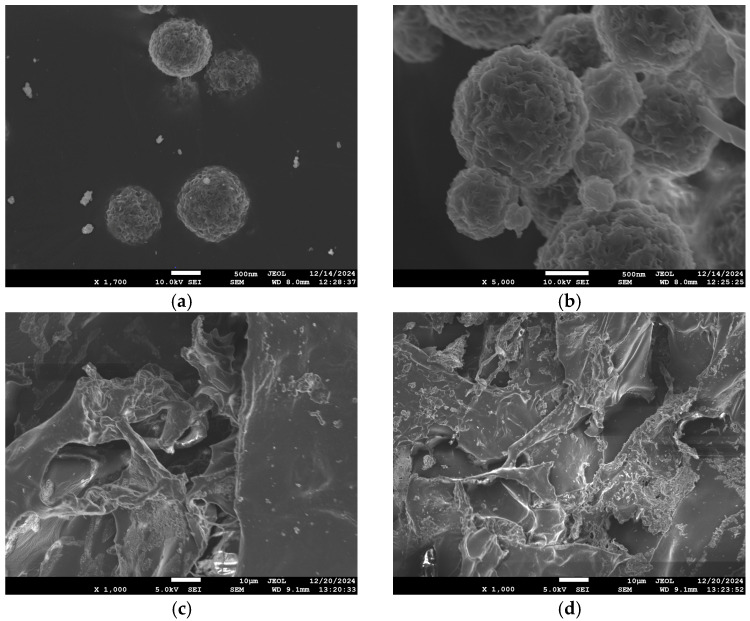
SEM micrographs of (**a**) neat PBSu nanoparticles, (**b**) Hep-loaded PBSu nanoparticles (PBSu-Hep), (**c**) CS-AMPS matrix with embedded NPs, and (**d**) CS-MES matrix with embedded NPs. Photograph courtesy of Despoina Meimaroglou. Copyright 2025.

The shape, size, and morphology of PBSu NPs, PBSu-Hep NPs, and the CS-MES and CS-AMPS wound dressing matrices were examined using SEM. The micrographs revealed overall highly porous and heterogeneous structures, as well as the successful integration of nanoparticles (NPs) into the polymeric matrices (Figure 6).

PBSu nanoparticles (Figure 6a) exhibited a spherical shape with relatively uniform size distribution, while the PBSu-Hep nanoparticles (Figure 6b) maintained a similar spherical morphology but appeared slightly aggregated and rougher, likely due to Hep loading altering surface characteristics. The rougher surface and variability in pore size suggest improved flexibility and greater potential for enhanced interaction with encapsulated or adsorbed agents, such as NPs or therapeutic molecules [49]. Furthermore, based on the scale bar in the SEM images (10 µm) and comparison with dynamic light scattering (DLS) measurements showing a hydrodynamic diameter of approximately 600–700 nm, the observed size range of the nanoparticles is in agreement with DLS data, confirming their nanoscale dimensions.

The modified CS derivatives (Figure 6c,d) showed distinct morphologies. CS-AMPS + NPs and CS-MES + NPs matrices displayed irregular structures composed of rough and smooth flake-like layers, with disrupted porous networks. Pore sizes ranged from 10 μm to over 100 μm, indicating that chemical modifications led to the breakdown of the original semi-crystalline order of chitosan, resulting in more amorphous and flexible structures. A similar structural transformation was also observed in a previous study by our group, where chitosan was modified with oxidized dextran (ODEX) via Schiff base reactions, leading to enhanced network formation and improved internal morphology [50].

The integration of PBSu NPs within these modified matrices highlights the synergistic potential of combining biodegradable nanoparticle systems with chemically tuned polysaccharide scaffolds. Such composites offer dual benefits: the porous polymeric matrix can support cell infiltration and nutrient diffusion, while the NPs can enable localized and sustained delivery of bioactive molecules like Hep.

### 3.3. Release Studies

The in vitro profiles of free Hep, Hep encapsulated in PBSu NPs, and Hep-loaded NPs incorporated into CS-based matrices (CS, CS-MES, CS-AMPS) were evaluated at pH = 7.4 to simulate physiological conditions. Table 2 includes the Hep loading efficiencies for each formulation. Hep was loaded 13.87 ± 1.06 in PBSu NPs, in line with other similar polymeric matrices in the literature [41]. The NPs retained their loading after incorporation in the CS matrices, since it was at least 10%, indicating that Hep remained entrapped during the dressing preparation.

**Figure 7:**
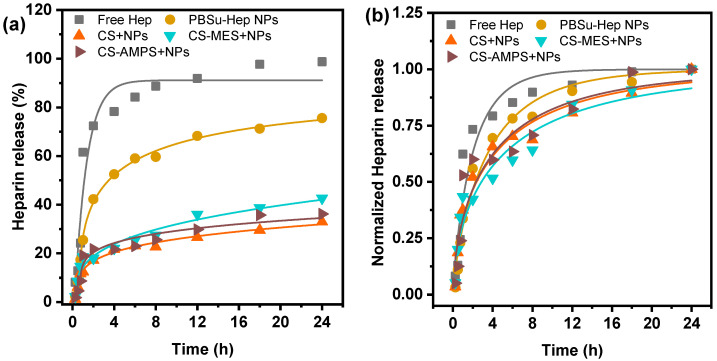
(**a**) Release profiles of free Hep, Hep encapsulated in PBSu NPs (PBSu NPs), and Hep-loaded NPs incorporated into CS-based matrices (CS, CS-MES, CS-AMPS) at pH 7.4. Hep release data were fitted using a modified Weibull function, which accounts for a delayed onset, a finite asymptotic release value and a non-exponential release profile. (**b**) Normalized release profiles of free Hep, Hep encapsulated in PBSu NPs, and Hep-loaded NPs incorporated into CS-based matrices (CS, CS-MES, CS-AMPS) at pH 7.4. Hep release data were fitted using a standard Weibull cumulative distribution function (CDF).

The in vitro release profiles are shown in Figure 7a. Free Hep exhibited a rapid burst release in PBS, with more than 70% of the Hep released within the first 2 h and approximately 98.8% by 24 h. This rapid release, in the absence of any diffusional barrier, poses a risk for dose dumping. The data were fitted using the modified Weibull function (Equation (10)) to describe Hep release kinetics. Consistently, its release profile corresponds to a high shape paremeter β-value of 0.87, indicative of near first-order kinetics, which implies a relatively unrestricted diffusion process with minimal barrier, i.e., the rate is proportional to the remaining unreleased Hep (Table 3). In contrast, Hep encapsulated in PBSu NPs showed a more controlled release pattern (Figure 7a). Following an initial burst release of 25.5% at 1 h which could be enhanced by the surface roughness of the NPs, the release progressed in a sustained manner, reaching 75.6% at 24 h. This sustained release pattern is governed primarily by diffusion, potentially modulated by physical relaxation of the PBSu matrix. Given the short release window (24 h) and the known slow degradation profile of PBSu, matrix degradation is expected to play a minimal role in this phase. The β-value of 0.81 supports this interpretation, as it implies a combined Fickian diffusion and Case II transport which could be the relaxation of PBSu [51]. While diffusion remains the dominant process, the scale parameter α increased from 2.02 to 3.56 confirming the slower release, due to the entrapment and interactions between PBSu and Hep (e.g., hydrogen bonding).

When the Hep-loaded NPs were incorporated into CS-based matrices (neat CS, CS-MES, CS-AMPS) a biphasic, highly sustained release was observed (Figure 7a). Initial release with the first 4 h ranged from 21.6 to 22.0%, likely due to surface-localized Hep or NPs near the matrix surface. This was followed by a diffusion-limited phase, with cumulative release at 24 h of 33% (CS), 36.2% (CS-AMPS) and 42.7% (CS-MES). These data suggest a synergistic release retardation effect from both the PBSu encapsulation and the CS matrix, facilitated by the encapsulation itself but also the interactions between Hep and CS [52]. Quantitatively, relative to free Hep, PBSu NPs reduced the 24 h release to 76%, and the CS-based matrix systems lowered it even further to 43–33%. The delay in release was reflected on the higher α-values of all formulations with CS matrices. The shape parameter β of the formulations with CS matrices were β ≤ 0.75 (Table 3), representing Fickian diffusion [51]. Therefore, the incorporation strategy produced a gradual shift in the dominant release mechanism—from near first-order kinetics (free Hep), to combined Fickian and Case II transport (PBSu-Hep NPs), and finally to mostly Fickian diffusion in the CS matrix systems.

It should be noted that, although amorphousness generally promotes diffusion-controlled release by providing more continuous, hydrated domains for solute transport, in our formulations the net heparin diffusion is additionally governed by strong electrostatic and hydrogen-bonding interactions between heparin and the functional groups of PBSu and the CS derivatives, which effectively reduce the apparent diffusion coefficient and lead to the observed sustained, Fickian release.

### 3.4. Biological Studies

#### 3.4.1. Biocompatibility Evaluation by MTT Assay

**Figure 8:**
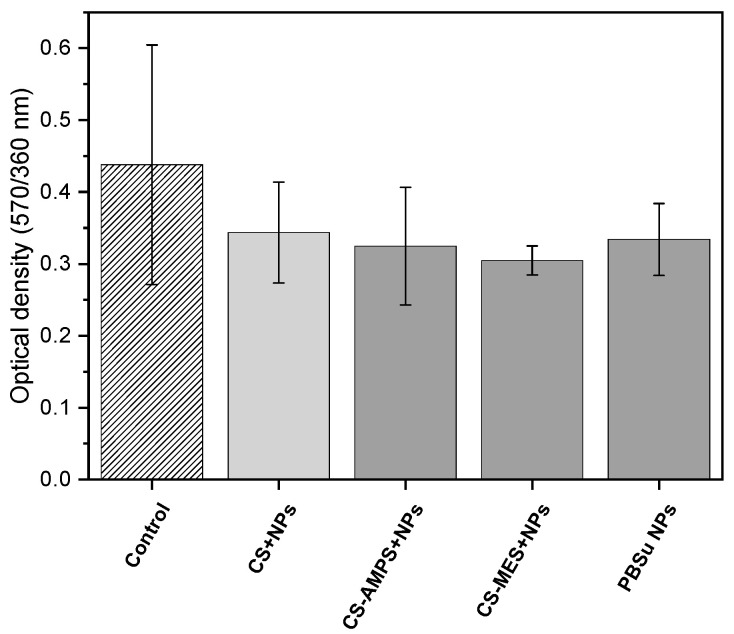
Evaluation of biocompatibility by MTT Assay for Control, CS + NPs, CS-AMPS + NPs, CS-MES and PBSu + Hep NPs.

The MTT assay data (Figure 8) demonstrated that fibroblast viability in the presence of all tested samples showed no statistically significant difference compared to the control group (cells cultured without samples). These findings confirm that the prepared scaffolds and nanoparticles exhibited satisfactory biocompatibility, maintaining high levels of cell viability. Moreover, the results suggest that biocompatibility was not affected by the release of Hep, as similar cell viability values were obtained for both Hep-loaded and unloaded nanoparticles. Overall, the materials were well tolerated by human dermal fibroblasts, supporting their potential for biomedical applications.

#### 3.4.2. Evaluation of Antibacterial Activity

**Figure 9:**
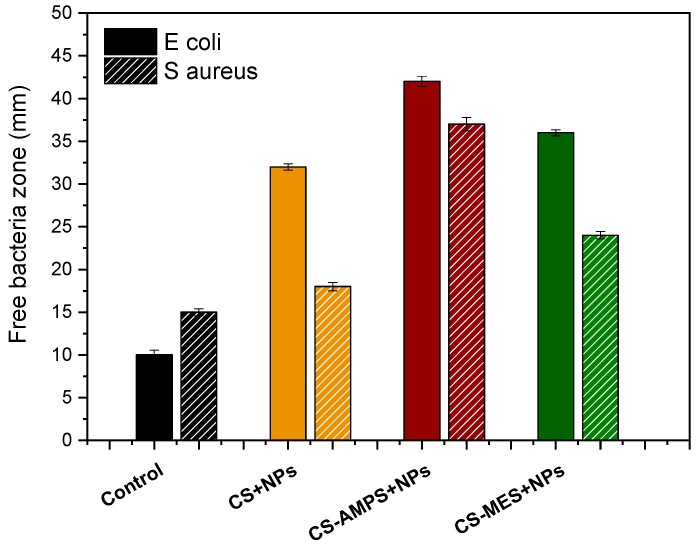
Antibacterial activity of control, CS + NPs, CS-AMPS + NPs and CS-MES + NPs.

The antibacterial activity of CS + NPs, CS-AMPS + NPs, and CS-MES + NPs against *S. aureus* and *E. coli* is presented in Figure 9. All the materials inhibited the growth of *E. coli*, with inhibition zones of approximately 32 mm for CS + NPs, 42 mm for CS-AMPS + NPs, and 36 mm for CS-MES-NPs compared to only ~10 mm for the control. This effect can be attributed to the cationic amino groups of chitosan, which electrostatically interact with the negatively charged bacterial cell walls, leading to membrane disruption and growth inhibition. In contrast, for the Gram-positive *S. aureus*, pristine CS + NPs exhibited only modest activity (~18 mm), while CS-AMPS + NPs demonstrated a significantly larger inhibition zone (~37 mm), and CS-MES + NPs a smaller at 24 mm. This enhanced activity is ascribed to the presence of the zwitterionic AMPS and MES groups, which provide both positive and negative charges to the polymer backbone. The dual charge distribution promotes stronger electrostatic interactions, allowing CS-AMPS + NPs and CS-MES + NPs to overcome the intrinsic resistance of positively charged Gram-positive bacterial surfaces. These quantitative results confirm that chemical modification of CS with AMPS broadens and enhances its antibacterial spectrum, making the developed material more versatile for potential wound-healing applications. Future studies will investigate the antibacterial performance of the samples under clinically relevant conditions, such as in protein-rich media or simulated wound fluid, to better mimic the in vivo wound environment.

#### 3.4.3. Determination of Blood Clotting Time (CT)

Blood clotting, or coagulation, is a crucial physiological process that prevents excessive bleeding by forming a fibrin clot through platelet activation and plasma protein interactions [53]. In this study, we evaluated whether blood clotting is affected by three different CS-based materials: CS + NPs, CS-MES + NPs, and CS-AMPS + NPs. The control for both determination of blood clotting time (CT) and determination of total hemostasis time (THT) and total blood loss (TBL) was a cotton sponge (Control), used as a conventional commercial dressing control, as it is widely employed in clinical practice and serves as a standard comparator in experimental hemostasis models. Cotton sponge promotes bleeding control primarily through blood absorption and mechanical compression rather than through active coagulation mechanisms. Therefore, its use as a reference material enables the evaluation of the enhanced hemostatic performance of the developed chitosan-based dressings.

**Figure 10:**
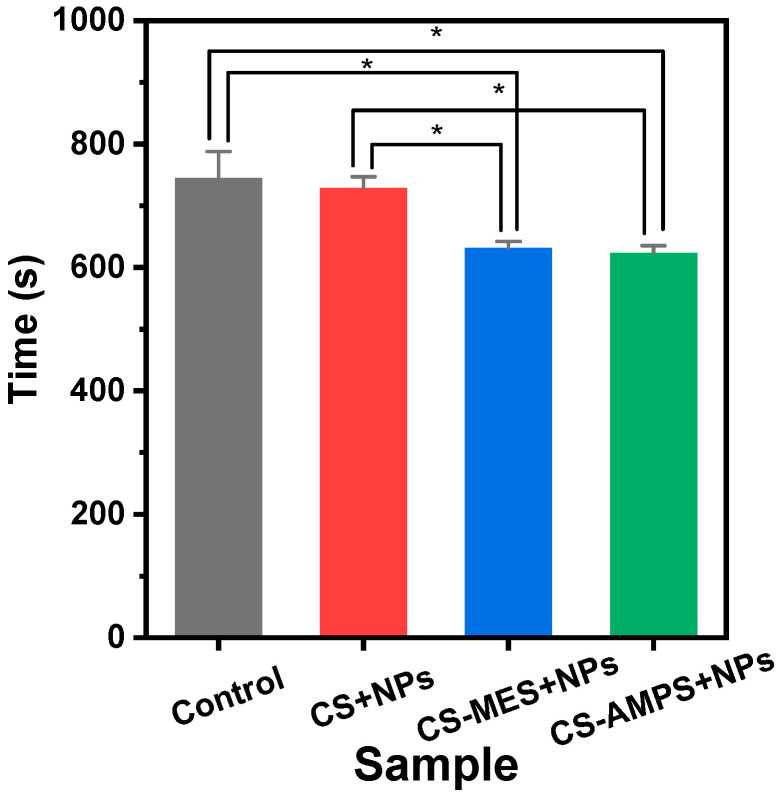
Blood clotting time of Control (cotton sponge), neat CS + NPs, CS-MES + NPs, and CS-AMPS + NPs. * *p* < 0.05.

The results demonstrated that CS-MES + NPs or CS-AMPS + NPs samples had significantly reduced CTs, with averages of approximately 632 and 623 s, respectively, compared to 745 s in the control group (*p* = 0.0021 and *p* = 0.0013; Figure 10). In contrast, no significant difference in CT was observed between the CS + NPs and the control group, indicating that the addition of PBSu-Hep NPs alone did not significantly affect coagulation; regardless, a decreasing trend in CT was present. Not surprisingly, significant differences were present between CS + NPs and CS-MES + NPs and between CS + NPs and CS-AMPS + NPs (*p* = 0.007 and *p* = 0.004; Figure 10). The reduction in CT with the use of CS-MES + NPs or CS-AMPS + NPs likely arises from the incorporation of sulfonic acid functional groups, which can interact with blood components to accelerate coagulation [54]. These modifications, through distinct mechanisms, may change surface charge and increase hydrophilicity, thereby promoting enhanced platelet adhesion and fibrin network formation. In particular, AMPS, with its strongly anionic sulfonate groups, increases the hydrophilicity of the chitosan matrix, enhancing plasma uptake and concentrating erythrocytes, platelets, and coagulation proteins at the wound interface to accelerate fibrin polymerization. MES on the other hand, bears a zwitterionic morpholino–sulfonate structure that forms a stable, highly hydrated interfacial layer, selectively supporting clot formation at the site of application whilst minimizing nonspecific platelet or protein activation elsewhere. In summary, CS-MES + NPs and CS-AMPS + NPs show promise for hemostatic applications such as wound dressings and biomedical devices that require rapid coagulation.

Within this context, our findings add to the recent research which has increasingly focused on the development of multifunctional or dual-functional wound dressings that combine hemostatic activity with therapeutic delivery. Several studies have reported chitosan-based materials incorporating antimicrobial agents, growth factors, or drug-loaded nanoparticles to simultaneously promote bleeding control and enhance wound healing. Compared with these systems, the present approach integrates heparin-loaded nanoparticles within a chitosan-based dressing, enabling both rapid hemostasis and the controlled release of a bioactive molecule. This dual functionality may enhance the therapeutic potential of the dressing by combining immediate bleeding control with localized pharmacological activity. However, further studies that include platelet activation assays and in vivo evaluations, are required to assess their effectiveness and safety profiles.

#### 3.4.4. Determination of Total Hemostasis Time (THT) and Total Blood Loss (TBL)

**Figure 11:**
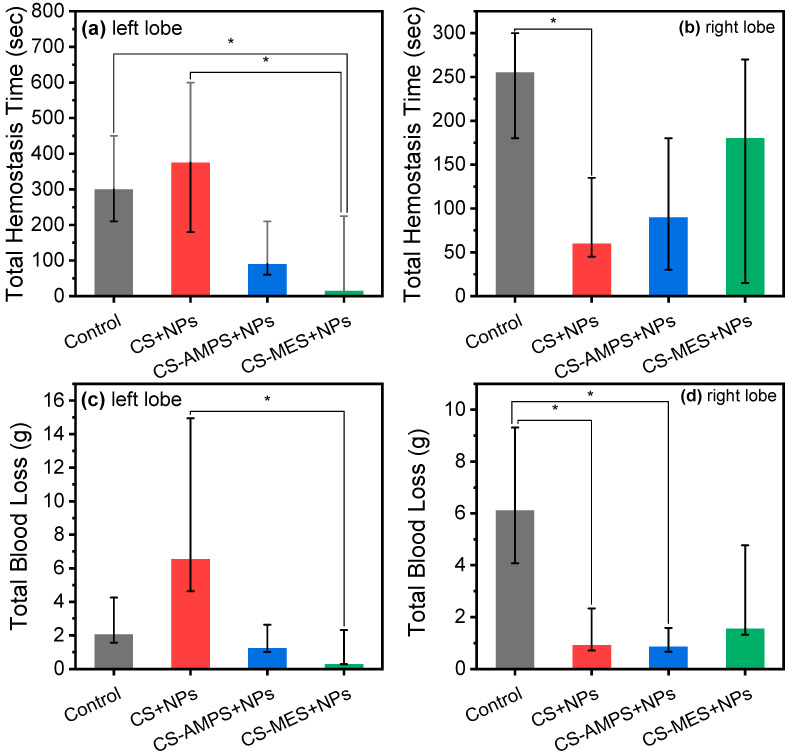
Total blood loss of Control (cotton sponge), CS + NPs, CS-MES + NPs, and CS-AMPS + NPs. * *p* < 0.05.

Total hemostasis time (THT) and total blood loss (TBL) measured in the different liver lobes are shown in Figure 11. In the present study, the Scheirer–Ray–Hare test showed no significant main effect of liver lobe on the THT. However, a significant interaction effect between liver lobe and dressing type was observed (*p* = 0.018), along with an overall effect of dressing type on THT (*p* = 0.003). This interaction may be attributed to known morphological differences in the vascular supply between the right and left liver lobes, particularly in portal and arterial vasculature [55,56].

Post hoc analyses revealed that in the left liver lobes, CS-MES + NPs dressings resulted in a significantly lower median THT, namely 15 s, compared to Control, i.e., 300 s (*p* = 0.022,) and between the former and CS + NPs (375 s, *p* = 0.031). In the right liver lobes, a significantly reduced THT was observed only with CS + NPs dressings compared to Control (60 s versus 255 s; *p* = 0.003).

Similarly, analysis of TBL showed no significant main effect of liver lobe, but a significant interaction effect between liver lobe and dressing type (*p* = 0.002), as well as an overall effect of dressing type (*p* = 0.005). As before, this interaction may be explained by differences in vascular morphology between liver lobes.

Post hoc comparisons demonstrated that in the left liver lobes, TBL was significantly lower with CS-MES + NPs dressings (0.2888 g) compared to CS + NPs dressings (6.5421 g; *p* = 0.03). In the right liver lobes, both CS-AMPS + NPs (0.8496 g; *p* = 0.012) and CS + NPs dressings (0.8496 g; *p* = 0.019) resulted in significantly lower TBL compared to Control (6.1151 g).

It should be noted that cotton, used here as a standard control dressing, exhibits very high capillarity and rapid wicking, which can provide immediate mechanical tamponade of bleeding. This effect may transiently reduce blood loss more effectively than CS + NPs in the left lobe, where vascular supply differs and bleeding can be more severe. By contrast, the hemostatic action of CS-based composites requires matrix wetting and charge-mediated aggregation of blood components and may be modestly delayed by the hydrophobic PBSu phase. Taken together with the known lobe-dependent anatomical and vascular variations in rabbits, these factors help explain the observed side-specific ranking. Importantly, across both lobes, the modified CS dressings (CS-MES + NPs and CS-AMPS + NPs) consistently outperformed the cotton control in reducing hemostasis time and total blood loss. This superior hemostatic performance is consistent with their swelling profiles: these formulations achieve high swelling ratios and water contents with slower water loss, maintaining a stable hydrogel layer at the wound interface during the critical early minutes of bleeding. This hydrated, cohesive matrix supports capillary uptake of blood and sustained presentation of charged functional groups, thereby facilitating platelet and erythrocyte capture and accelerating clot formation, whereas neat CS, with lower equilibrium swelling and faster deswelling, provides a less persistent hydrophilic environment for these interactions.

These findings align with a growing body of evidence supporting the efficacy of CS-based topical hemostatic agents, which have been increasingly investigated over the last two decades [57]. Various CS derivatives with different chemical modifications have demonstrated promising hemostatic performance in multiple in vivo wound and tissue models. For example, freeze-dried CS-based dressings effectively controlled severe parenchymal and venous hemorrhage in a swine liver trauma model [58]. Polysaccharide-modified CS significantly reduced blood loss and hemostasis time compared to pristine CS, cellulose nanocrystals, and Celox in mouse tail vein and rabbit femoral artery wounds [59]. Hemostasis times in those studies ranged from approximately 75 ± 1.63 sec for CS hydrogel in rabbit liver wounds to 163.3 ± 5.8 sec in rabbit femoral artery wounds, and 209 ± 81 sec in rabbit lingual wounds [59,60,61].

Our observed THT values following CS-MES + NPs, CS-AMPS + NPs, and CS + NPs dressing application in the right Hepatic lobe, namely 180, 90, and 60 sec, compare favourably with these prior results. For example, a protonated CS derivative was recently tested using rabbit livers and the blood loss was reduced about 50%, while clotting time was reduced about 10% in comparison to Celox [62]. Herein, compared to neat CS, CS-MES + NPs reduced THT by 96% in the left liver lobe, while CS + NPs reduced it by 84% in the right lobe. Regarding TBL, CS-MES + NPs achieved a 96% reduction in the left lobe, and both CS-AMPS + NPs and CS + NPs achieved 86% reductions in the right lobe. These improvements highlight the superior efficacy of the modified, hep-containing CS-based dressings, arising from the synergistic combination of chemical modification with sulfonic/sulfonyl groups, making CS more hydrophilic and promoting electrostatic interactions with blood platelets and proteins, and controlled anticoagulant (Hep) release. Nevertheless, direct quantitative comparisons between studies remain challenging due to differences in chitosan material formulations, wound types, tissue models, and methodologies [59,60].

## 4. Conclusions

The study investigated the structural, physicochemical, and hemostatic properties of modified chitosan-based dressings enriched with Hep-loaded poly(butylene succinate) (PBSu) nanoparticles (NPs) for wound care applications. The dressings were then systematically characterized and evaluated using a combination of techniques. FT-IR spectroscopy confirmed the successful chemical modifications of chitosan with MES and AMPS, as well as the effective encapsulation of Hep within the PBSu NPs. XRD patterns revealed a decrease in chitosan crystallinity upon modification, with further structural changes evident in the porous morphology observed via SEM. Swelling studies highlighted enhanced water uptake and stability in the case of the modified dressings compared to neat CS under neutral pH, while acidic conditions led to a premature network collapse. MTT assay and antibacterial activity measurements demonstrated that the developed scaffolds and NPs exhibit both excellent cytocompatibility and strong antibacterial activity, highlighting their dual functionality. Collectively, the results support their promising potential as advanced biomaterials for wound-healing and broader biomedical applications. In vitro release studies demonstrated that Hep release was progressively sustained across formulations: from rapid, near first-order release for free Hep, to slower, diffusion-controlled release from PBSu NPs, and finally to biphasic, highly sustained profiles in CS-based matrices. Weibull modelling confirmed a shift from near first-order kinetics (β = 0.87) to Fickian diffusion (β ≤ 0.75), supported by increasing α-values. Moreover, the hemostatic performance was significantly improved, with clotting times reduced compared to neat CS, indicating that these formulations can enhance hemostasis while maintaining anticoagulant activity. From a translational standpoint, the further development of this dual-functional hemostatic/anticoagulant dressing will require addressing key challenges that have also been highlighted for other complex multifunctional dressings, including scalable and reproducible manufacture of nano-engineered and chemically modified components, compatibility with clinically relevant sterilization methods (e.g., γ-irradiation or ethylene oxide), and alignment with regulatory frameworks for combination products that provide both hemostatic and pharmacologically active functions. Systematic studies on long-term safety, manufacturability, and regulatory positioning will therefore be essential to advance this platform toward clinical application [63,64,65].

## Figures and Tables

**Table 1:** Swelling kinetics fit results using a first-order kinetics model (Equation (8)).

Sample	Maximum Water Content (%)	Water Content Loss (%)	k (g/g∙min)	R^2^
CS	94.03	13.27	4.6 × 10^−3^	0.93
CS-MES	95.73	4.34	1.4 × 10^−3^	0.90
CS-AMPS	98.04	5.23	0.83 × 10^−3^	0.92
CS-MES + NPs	94.03	9.24	2.7 × 10^−3^	0.94
CS-AMPS + NPs	91.73	9.37	3.3 × 10^−3^	0.92

**Table 2:** Particle size and PDI values of the prepared PBSu NPs and loading of the Hep-loaded NP formulations (%, *w*/*w*).

Sample	Z-Average (nm)	PDI	Hep Loading %
PBSu NPs	506	0.39	-
PBSu-Hep NPs	797	0.58	13.87 ± 1.06
CS + NPs	-	-	10.24 ± 1.85
CS-AMPS + NPs	-	-	12.43 ± 0.97
CS-MES + NPs	-	-	11.29 ± 2.41

**Table 3:** Hep release efficiency comparison (24 h) and Weibull values calculated from the normalized data fit (Figure 7b).

Formulation	Release Efficiency at 24 h (%)	Relative to Free Hep	Weibull α-Values (Normalized Fit)	Weibull β-Values (Normalized Fit)
Free Hep	98.8	×1.00	2.02	0.87
PBSu-Hep NPs	75.6	×0.76	3.56	0.81
CS + NPs	33	×0.33	4.34	0.61
CS-MES + NPs	42.7	×0.43	5.30	0.60
CS-AMPS + NPs	36.2	×0.37	4.18	0.63

## Data Availability

The original contributions presented in this study are included in the article. Further inquiries can be directed to the corresponding authors.
